# Role of immunomodulatory probiotics in alleviating bacterial diarrhea in piglets: a systematic review

**DOI:** 10.1186/s40104-024-01070-z

**Published:** 2024-08-12

**Authors:** Sudeb Saha, Fu Namai, Keita Nishiyama, Julio Villena, Haruki Kitazawa

**Affiliations:** 1 Food and Feed Immunology Group, Laboratory of Animal Food Function, Graduate School of Agricultural Science, Tohoku University, Sendai, 980-8572 Japan; 2 Department of Dairy Science, Faculty of Veterinary, Animal and Biomedical Sciences, Sylhet Agricultural University, Sylhet, 3100 Bangladesh; 3 Livestock Immunology Unit, International Education and Research Center for Food and Agricultural Immunology (CFAI), Tohoku University, Sendai, 980-8572 Japan; 4 Laboratory of Immunobiotechnology, Reference Centre for Lactobacilli (CERELA-CONICET), 4000 Tucuman, CP Argentina

**Keywords:** *Clostridium*, Diarrhea, *E. coli*, Immunomodulatory effect, Piglets, Probiotics

## Abstract

Diarrhea is a common enteric disease in piglets that leads to high mortality and economic losses in swine production worldwide. Antibiotics are commonly used to prevent or treat diarrhea in piglets. However, irrational antibiotic use contributes to the development of resistance in bacteria and antibiotic residues in animal products, threatening public health, while causing gut microbiota dysbiosis and antibiotic-resistant bacterial infection in piglets. Therefore, the quest for alternative products (such as probiotics, prebiotics, organic acids, enzymes, essential oils, medium-chain fatty acids, zinc, and plant extracts) has recently been clearly emphasized through the increase in regulations regarding antibiotic use in livestock production. These antibiotic alternatives could lower the risk of antibiotic-resistant bacteria and meet consumer demand for antibiotic-free food. Several antibiotic alternatives have been proposed, including immunomodulatory probiotics, as candidates to reduce the need for antimicrobial therapy. Many studies have revealed that probiotics can avert and cure bacterial diarrhea by regulating the gut function and immune system of piglets. In this review, we focus on the major pathogenic bacteria causing piglet diarrhea, the research status of using probiotics to prevent and treat diarrhea, their possible mechanisms, and the safety issues related to the use of probiotics. Supplementation with probiotics is a possible alternative to antibiotics for the prevention or treatment of bacterial diarrhea in piglets. Furthermore, probiotics exert beneficial effects on feed efficiency and growth performance of piglets. Therefore, appropriate selection and strategies for the use of probiotics may have a positive effect on growth performance and also reduce diarrhea in piglets. This review provides useful information on probiotics for researchers, pig nutritionists, and the additive industry to support their use against bacterial diarrhea in piglets.

**Graphical Abstract:**

Interaction of probiotics with the gut associated immune system. TLRS, Toll-like receptors; MAPK, Mitogen-activated protein kinases; TRAF, Tumor necrosis factor receptor-associated factor; DC, Dendritic cells; MP, Macrophages; NT, Naïve T cell; IL-10, Interleukin 10 proteins; Tregs, Regulatory T cells; Th1, Type 1 T helper cells; Th2, Type 2 T helper cells; Th17, Type 17 T helper cells; SIgA, Secretory immunoglobulin A; TJs, Tight junctions.

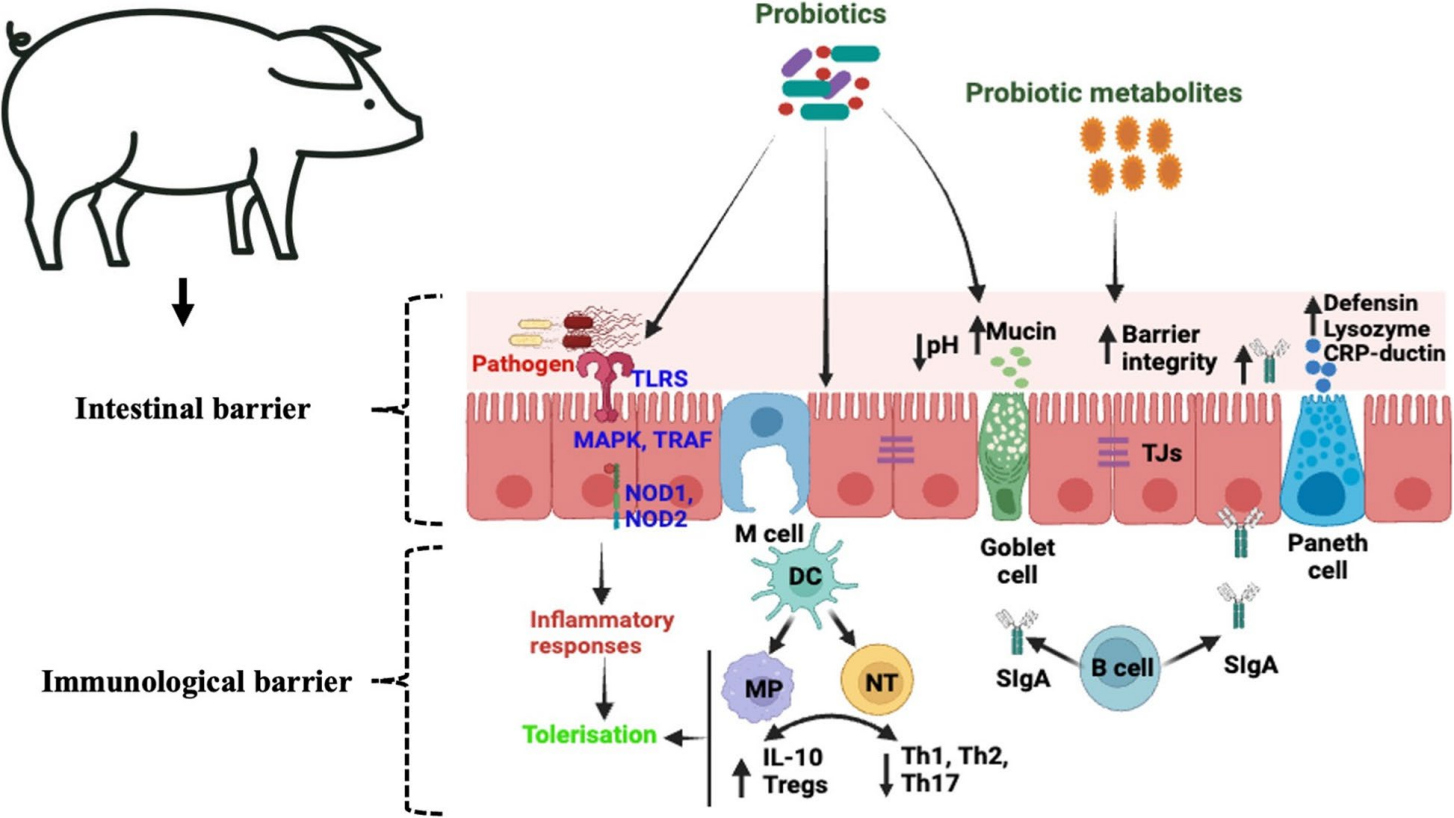

## Introduction

Diarrhea is the most prevalent enteric disease in modern pig production and leads to high mortality, reduced growth rates and increased treatment costs [1, 2]. Neonatal diarrhea occurring throughout the suckling piglet period and post-weaning diarrhea occurring within the first two weeks after weaning are the two most prominent forms of diarrhea in piglets [3, 4]. After birth, the mucosal immune system of piglets is immature. As a result, pathogens can easily colonize the intestinal tract, leading to the development of neonatal diarrhea. Moreover, the weaning process causes stress in piglets and thus impairs intestinal function, which allows pathogens to develop post-weaning diarrhea. The mortality rate of piglets with diarrhea can increase by up to 75% depending on the severity of diarrhea and the age of piglets [5]. The pre-weaning mortality rate ranges from 10% to 20% in the main pig-producing countries [6], whereas mortality can reach up to 25% due to diarrhea during the post-weaning period [2]. Diarrhea outbreaks in piglets are caused by different enteropathogens such as bacteria, viruses, protozoa, and parasites [7]. Furthermore, non-infectious factors, such as poor nutrition, management and stress, can lead to an increased risk of enteropathogenic infection which causes diarrhea in piglets.

Recently, diarrhea in piglets, particularly that of infectious origin, has regained attention because approximately half (49%) of piglet deaths result from diarrhea infections that cause severe economic losses in the swine industry worldwide [8]. Diarrhea due to bacterial infection is common in piglets. Among the bacteria that cause diarrhea in piglets, the main pathogens are *Escherichia coli*, *Salmonella* spp. and *Clostridium* spp. [9]. Antibiotics are commonly used in the modern pig industry [10]. However, the long-term and overuse of antibiotics in animal diets has led to drug-resistant bacteria in animals and humans [11, 12]. Thus, antibiotics used in animals were banned in the European Union in 2006 [13] or allowed for limited use in many countries such as USA and Japan [11, 14]. Moreover, the use of antibiotics in feed production enterprises to commercial feed as growth promoters in pig production has been banned in China from 2020 [15]. In this context, researchers, farmers, and the pig industries are making substantial efforts to find new alternatives to antibiotics in pig farming. Thus, probiotics, prebiotics, organic acids, enzymes, essential oils, medium chain fatty acids, zinc, and plant extracts have been used as alternatives to antibiotics in in vitro and in vivo studies [16]. Among them, probiotics have been tested or used as a replacement for antibiotics because they can decrease the pathogen load and alleviate gastrointestinal disorder symptoms by restoring the microbial balance in animals [17]. Many of the gut microbes can play immunomodulatory roles in the host. The most frequently used probiotic microorganisms are *Lactobacillus*, *Bifidobacterium*, *Enterococcus*, *Bacillus*, *Pediococcus* and yeast genera *Saccharomyces* for treating diarrhea in piglets as it has recently been well documented in recent years (Tables 1 and 2). Some probiotic strains confer immunological protection to the host by modulating the immune response [18]. These probiotic strains are designed as “immunobiotics” [19]. Thus, exploring and gaining knowledge of the interactions among immunomodulatory probiotics, pathogens, and the host’s gastrointestinal tract during diarrhea in piglets will help develop new probiotics (immunobiotics) that can help to protect animals from diarrhea and enhance growth performance.

**Table 1:** Use of probiotics against bacterial pathogens in in vitro infection models

Strain	Experimental design	Host/Experimental mode	Physiological effect	Immunomodulatory effect	References
*L. jensenii* TL2937 *L. reuterii* MEP221102 *L. rhamnosus* MEP221111	*L. jensenii* TL2937 *L. reuterii* MEP221102 *L. rhamnosus* MEP221111 (5 × 10^7^ cells/mL) Period: 48 h	PIE cells ETEC strain 987P (O9: H−: 987 pilus + : heat stable toxin +) challenge (5 × 10^7^ cells/mL) and LPS challenge (1,000 ng/mL; from *E. coli* O55:B5)	-	After 24 h ETEC challenge: ↑ *IL-1α* mRNA in PIE cells ↓ *IL-8* mRNA in PIE cells ↓ *MCP-1* mRNA expression in PIE cells for MEP221102 After 48 h ETEC challenge: ↓ IL-6 and IL-8 proteins LPS challenge: ↓ *IL-6, IL-8*, and *MCP-1* mRNA in PIE cells for TL2937 ↑ Phosphorylation of p38, ERK, and JNK in PIE cells for TL2937	Shimazu et al. (2012) [20]
*B. longum* BB536; *B. breve* M-16 V and MCC-1274; *B. infantis* MCC 12; *B. pseudolongum* MCC-92; *L. paracasei* MCC-1375; *L. gasseri* MCC-1183 and MCC-587; *L. lactis* MCC-866 and MCC-1723; and *L. helveticus* MCC-648	*B. longum* BB536; *B. breve* M-16 V and MCC-1274; *B. infantis* MCC-12; *B. pseudolongum* MCC-92; *L. paracasei* MCC-1375; *Lactobacillus gasseri* MCC-1183 and MCC-587; *L. lactis* MCC-866 and MCC-1723; and *L. helveticus* MCC-648 (5 × 10^7^ cells/mL) Period: 12 h	PIE cells Heat-stable ETEC PAMPs challenge (5 × 10^7^ cells/mL)	-	↓ IL-6, IL-8 and MCP-1expressions in PIE cells for *Bifodobacterium longum* BB536 and *Bifidobacterium breve* M-16 V strains	Tomosada et al. (2013) [21]
*L. jensenii* TL2937	5 × 10^7^ cells/mL Period: 5 d	PIE-APCs co-cultures ETEC challenge in PIE cells (5 × 10^7^ cells/mL for 12 h)	-	↑ Expression of MHC-II, CD80/86, IL-10, and Bcl-3 in CD172a + CD11R1 − and CD172a + CD11R1 adherent cells by *L. jensenii* TL2937 ↓ *IL-6* and *IL-8* in PIE	Suda et al. (2014) [22]
*L. delbrueckii *subsp.* delbrueckii *TUA4408L	*L. delbrueckii* TUA4408L (5 × 10^7^ cells/mL) Period: 12 h	PIE cells ETEC challenge (5 × 10^7^ cells/mL)	-	↓ MAPK and NF-κB activation ↓ *IL-6*, *IL-8*, and *MCP-1* in PIE cells	Wachi et al. (2014) [23]
*L. reuteri* LR1	*L. reuteri* LR1 (10^8^ CFU/mL) Period: 12 h	IPEC-1 cells ETEC O149:K91, K88ac challenge (10^8^ CFU/mL)	↑ Adhesion to IPEC-1 cells ↓ Adhesion ETEC to IPEC-1 cells	↓ ETEC-induced expression of proinflammatory transcripts *IL-6* and *TNF-α* and protein IL-6 in IPEC-1 cells ↑ Anti-inflammatory cytokine *IL-10* in IPEC-1 cells	Wang et al. (2016) [24]
*L. rhamnosus* GG	*L. rhamnosus* GG (3 × 10^8^ CFU) Period: 12 h	IPEC-J2 cells *S. enterica* serovar Infantis challenge (3 × 10^8^ CFU, MOI 600:1)	↓ Invasion *S.* Infantis invasion in the IPEC-J2 cells	↓ *S.* Infantis-induced autophagy ↑ Epidermal growth factor receptor and Akt phosphorylation	Zhang et al. (2018) [25]
*L. johnsonii* LJ202 *L. reuteri* LR108	*L. johnsonii* LJ202 *L. reuteri* LR108 (10^6^ CFU/mL) Period: 24 h	Co-culture *S. enterica *serovar Enteritidis DMST7106 and coliform (10^3^ CFU/mL)	Exhibits inhibitory activity against *Salmonella* spp. and fecal coliform bacteria	-	Abhisingha et al. (2018) [26]
*L. salivarius* isolates	*L. salivarius* (10^6^ CFU/mL) Period: 12 h	PIE cells ETEC strain 987P (O9: H−: 987 pilus + : heat stable toxin +) challenge (3 × 10^7^ cells/mL)	-	↓ *IL-6* expression in PIE cells	Masumizu et al. (2019) [27]
*L. fermentum *UCO-979C *L. fermentum *CRL973	*L. fermentum *UCO-979C (5 × 10^8^ cells/mL) Period: 12 h	PIE cells ETEC strain 987P (O9: H−: 987 pilus + : heat stable toxin +) challenge (5 × 10^7^ cells/mL)	-	*L. fermentum* UCO-979C effect: ↑ *CXCL9*, *CCL8* expression in PIE cells ↓ *CXCL8*, *CXCL-10*, *CXCL-11* in PIE cells *L. fermentum *CRL973effect: ↓ *CXCL5* expression in PIE cells	Garcia-Castillo et al. (2019) [28]
*L. salivarius* FFIG35, FFIG58	*L. salivarius* (5 × 10^7^ cells/mL) Period: 5 d	PIE cells ETEC (5 × 10^6^ cells/mL)/ Rotavirus (Rotavirus strain containing 1 μg/mL of trypsin) challenge	-	↑ *IFN- β*, *IFN-λ* and antiviral factors in PIE cells	Indo et al. (2021) [29]
*B. subtilis* (CP9)	*B. subtilis* CP9 (10^8^ CFU/well) Period: 24 h	IPEC-J2 cells *E. coli* (ETEC), serotype K88 challenge (10^8^ CFU/well)	↓ Intestinal inflammation	↓ Apoptosis ↑Cell proliferation, possibly by metabolic modulation, ↑Anti-inflammatory granulocyte–macrophage colony stimulating factor, host defense peptide mucin 1 ↑ Epithelial barrier function ↓ mRNA expression of *TLR2*, *TLR4* and *TLR9* in IPEC-J2 cells ↓ *TNF-α*, *IL-6*, *IL-8*	Sudan et al. (2022) [30]
*L. plantarum* CRL1506, CRL681	*L. plantarum* CRL1506, CRL681 (10^8^ cells/mL) Period: 12 h	PIE cells ETEC PAMPs 987P (O9: H−: 987 pilus + : heat stable toxin +) challenge (5 × 10^7^ cells/mL)	-	↓ Expressions of IL-8, *CCL2*, *CXCL5* and *CXCL9* in PIE cells for CRL1506 and CRL681 strains ↓ *A20* and *Bcl-3* for CRL 1506 ↑ *MKP-1* in PIE cells for CRL1506	Baillo et al. (2022) [31]

Abbreviations: ↑ Increased, ↓ Decreased, *PIE* Porcine intestinal epitelial cell line, *IPEC* Intestinal porcine epitelial cell line, *ETEC* Enterotoxigenic *Escherichia coli*, *MCP-1* Monocyte chemoattractant protein 1, *ERK* Extracellular signal regulated kinase, *JNK* c-JUN N-terminal kinase, *IL* Interleukin, *MHC* Major histocompatibility complex, *CD* Cluster of differentiation, *MAPK* Mitogen-activate protein kinase, *TNF* Tumor necrosis factor, *CXCL* Chemokine (C-X-C motif) ligand, *IFN* Interferon

**Table 2:** Use of probiotics against bacterial pathogens in in vivo infection models

Strain	Experimental design	Host/Experimental mode	Physiological effect	Immunomodulatory effect	References
*L. plantarum* Lq80	7.71 log CFU/g Route: Oral Period: 14 d	*22 crossbred (Landrace × Large white × Duroc) piglets	↓ Number of *E. coli*, *C. perfringens* and α toxin gene in feces or intestinal content ↑growth indigenous lactobacilli	-	Takahashi et al. (2007) [32]
*L. acidophilus* KCTC 3111 *L. plantarum* KCTC 3104 *B. subtilis* KCTC 3239 *S. cerevisiae* KCTC 7915	*L. acidophilus* (3.2 × 10^8^ CFU/g), *L. plantarum* (2.2 × 10^8^ CFU/g), *B. subtilis* (4.5 × 10^9^ CFU/g) *S. cerevisiae,* (5.2 × 10^8^ CFU/g) 0.5% in diet Route: Oral Period: 28 d	*72 crossbred (Landrace × Yorkshire) finishing pigs	↓ Feed conversion ratio ↑ Weight gain ↓ Fat and crude protein in meat ↓ Thiobarbituric acid value	*↑ TNF-α,* IL-6 in spleen cells	Ko et al. (2008) [33]
*S. cerevisiae* (XPC, Diamond V)	0.2% diet Route: Oral Period: 35 d	40 weaned piglets (premium genetics 1020, VA) *Salmonella* Nal^R^Nov^*R*^ challenge (5 mL oral dose of trypticase soy broth contain 10^9^ CFU of *S.* Typhimurium)	↑ Body weight ↑ Beneficial bacteria in the gastrointestinal tracts	-	Price et al. (2010) [34]
*L. plantarum* CJLP243	10^8^ CFU/kg 10^9^ CFU/kg 10^10^ CFU/kg Route: Oral Period: 28 d	108 crossbred piglets (Duroc × Yorkshire × Landrace) ETEC challenge (oral dose, 5 × 10^9^ CFU)	↑ Growth and health performance, body weight	↓* TNF-α,* IL-6 and IFN-γ in serum	Lee et al. (2012) [35]
*B. cereus* var. Toyoi	Sows: 3.14 × 10^5^ CFU/g *B. cereus* var. Toyoi Piglets: 8.7 × 10^5^ CFU/g *B. cereus* var. Toyoi (d0-d27) 6.5 × 10^5^ CFU/g *B. cereus* var. Toyoi d28 Route: Oral Period: 28 d	8 sows and 24 piglets (Landrace pure) *S****. ***Typhimurium DT104 challenge (Oral dose, 3 × 10^9^ CFU/piglet)	↑ Health status of piglets	↓ CD8 + γδ T cells in the peripheral blood and the jejunal epithelium	Scharek- Tedin et al. (2013) [36]
*L. plantarum* JC1 (B2028)	*L. plantarum* (2 × 10^10^ CFU/d), 20 mL/pig/d Route: Oral Period: 18 d	72 crossbred [(Large White × Landrace) × Pietrain] weanling piglets ETEC K88 challenge (6 mL oral dose, 2 × 10^9^ CFU/mL)	↓ Diarrhea ↑ Villous height and goblet cell in intestine	↓ TNF-α in serum	Guerra- Ordaz et al. (2014) [37]
*L. jensenii* TL2937	3. × 10^8^ CFU/g 200 g/d Route: Oral Period: 14 weeks	20 Cross piglets: Landrace × Large Yorkshire × Duroc	↑ Growth performance, productivity ↑ Body weight	↓ C reactive protein concentrations in plasma No changes in blood leukocytes, ratio of granulocytes to lymphocyte numbers, macrophages’ activity, and antibody levels	Suda et al. (2014) [22]
*S. cerevisiae* CNCMI-4407	5 g/kg live yeast *S. cerevisiae* 10^10^ CFU/g Route: Oral Period: 14 d	34 crossbred (30 piglets and 4 sow) Duroc × Pietrain × Landrace sow and piglets challenged with ETEC F4 O149:K88 (oral dose of 1.5 × 10^11^ CFU/piglet)	↓ Diarrhea (scores, duration and shedding of pathogenic ETEC bacteria in feces) ↑ Growth performance in piglets	↑ IgA in serum	Trckova et al. (2014) [38]
*L. plantarum* CGMCC1258	5 × 10^10^ CFU/kg diet Route: Oral Period: 18 d	72 male young piglets (Duroc × Landrace × Large White) ETEC K88 challenge (oral dose, 1 × 10^8^ CFU/piglet)	↑ Growth performance in piglets ↓ Diarrhea	↑ Intestinal barrier by protecting intestinal morphology and permeability ↑ expression of *ZO-1* and *occludin*	Yang et al. (2014) [39]
*B. subtilis* KN-42	2 × 10^9^ CFU/kg feed 4 × 10^9^ CFU/kg feed 20 × 10^9^ CFU/kg feed Route: Oral Period: 28 d	*360 crossbred piglets (Duroc × Landrace × Yorkshire)	↑ Growth performance ↑ Avearge daily gain ↓ Diarrhea index ↓ Number of *E. coli* in feces	-	Hu et al. (2014) [40]
*L. reuteri* and *L. plantarum* complex	1 × 10^9^ CFU/kg, 0.1% diet Route: Oral Period: 28 d	*168 weanling crossbred pigs [(Yorkshire × Landrace) × Duroc]	↓ Fecal gas emission, ↓ Diarrhea score ↓ *E. coli* concentration in feces	-	Zhao and Kim (2015) [41]
*B. licheniformis* DSM 5749 *B. subtilis* DSM 5750	Low: 3.2 × 10^9^ CFU/mL High: 3.9 × 10^9^ CFU/mL Route: Oral Period: 15 d	32 male F4ab/acR^−^ crossbred (Landrace × Large White) piglets F4^+ ^ETEC challenge (10 mL oral dose, 10^9^ CFU/mL)	-	Low: ↑ *IL-6, TNF-α*, *IL-10 *still showed in the intestine after ETEC challenge Low or High: ↑ Percentage of Foxp3^−^IL-10^+^ T High: ↑ Generation of CD4^+^IL-10^+^ T cells in the intestine ↑ *T-bet* mRNA expression in the jejunum	Zhou et al. (2015) [42]
*L. plantarum* B2984	10^10^ CFU/animal/d Route: Oral Period: 17 d	24 mixed sexes piglets (Large white × Landrace) *S.* Typhimurium SL1344 challenge (10 mL oral dose, ~ 1 × 10^8^ CFU)	-	↑ Serum immunoglobulins (IgG, IgM, IgA)	Naqid et al. (2015) [43]
*B. licheniformis* DSM 5749 *B. subtilis* DSM 5750	Low: 10^8^ CFU/d High:10^8^ CFU/d Route: Oral Period: 15 d	32 mixed sex *MUC4* RR crossbred (Landrace × Large White) piglets F4^+^ ETEC challenge (10 mL oral dose, 1 × 10^10^ CFU)	↑ Epithelial barrier integrity	↑ CD4^−^CD8^−^ T-cell ↑ Intestinal cytokines (*IL-22*) ↑ Intestinal mRNA expression of *IκBα*, *TLR4*, *NOD2*, and *IL-8* ↑ Jejunal ZO-1 expression	Yang et al. (2016) [44]
*L. reuteri* ZLR003	2.0 × 10^9^ CFU/mL; 5 mL/piglet/d Route: Oral Period: 10 d	*9 Crossbred piglets (Landrace × Large White)	↑ Beneficial microbes in the gut as indicated by ACE and Chao 1 index	-	Zhang et al. (2016) [45]
Commercially available *Lactobacillus* spp. (GNc, Pennsylvania, USA)	2 × 10^6^ CFU *Lactobacillus* spp. Route: Oral Period: 72 h	150 piglets *C. difficile* challenge (Intragastrically 1.25 mL dose, *C. difficile* spores, 2 × 10^6^)	↓ Mesocolonic edema No clear effect on disease control	-	Arruda et al. (2016) [46]
*B. longum* subsp. *infantis *CECT 7210 *B. animalis *subsp*. lactis *BPL6	2 mL/animal 10^9^ CFU/g Route: Oral Period: 16 d	72 male piglets (Large White × Landrace) *S.* Typhimurium challenge (2 mL oral dose, 5 × 10^8^ CFU)	↓ Diarrhea ↓ Rectal temperature ↓ Pathogen shedding ↑ Feed intake ↑ Healthy fermentation profile	↑ Intestinal intraepithelial lymphocytes	Barba-Vidal et al. (2017) [47]
*S. cerevisiae* CNCM I-4407	5 × 10^10^ CFU/kg Route: Oral Period: 17 d	20 mixed sex piglets ETEC O149:F4ac challenge (1.5 mL oral dose, 10^8^ CFU)	↓ Impairment of intestinal mucosa Modulation of the transcriptomic profile of the intestinal mucosa	↑ A cluster of genes related to leukocyte, lymphocyte, and T cell activation in the intestinal mucosa	Trevisi et al. (2017) [48]
*L. casei* (No. 1.570) *E. faecalis* (No. 1.2024)	Ratio of 3:1 (*L. casei: E. fecalis*) 10^9^ CFU/mL Route: Oral Period: 28 d	*120 newborn suckling piglets (Duroc × Landrace × Yorkshire)	↑ Growth and health performance ↑ Microbial similarity coefficients in intestine ↓ Mortality, diarrhoea rates	↑ Villus length and the expression level of *TGF-α, β* in the jejunum, ↑IgA, IgG in plasma ↓ Jejunal *TNF-α*	Liu et al. (2017) [49]
*L. rhamnosus* GG	(1 × 10^9^ CFU/mL, 10 mL/d) Route: Oral Period: 18 d	21 pigs *S. enterica *serovar Infantis challenge (10 mL oral dose, 5 × 10^10^ CFU/mL)	↑ Gut microbiota balance ↓ infection of *S.* Infantis	↓ *S.* Infantis -induced autophagy in ileum ↑ Epidermal growth factor receptorand Akt phosphorylation in ileum	Zhang et al. (2018) [25]
*L. frumenti* JCM11122	*L. frumenti* (2 mL, 10^8^ CFU/mL) Route: Oral Period: 26 d	100 crossbred piglets (Landrace × Yorkshire)	↑ Fatty acids and protein metabolism ↓ Diseases-associated metabolic pathways ↑ Health promoting microbes in the gastrointestinal tracts	↑ IgG in serum, ↑ IgA, IFN-γ in intestinal mucosa ↑ Intestinal tight junction proteins (ZO-1, occluding and claudin)	Hu et al. (2018) [50]
*L. delbrueckii* CCTCCM207040	*L. delbrueckii* (5 × 10^8^ CFU/mL) 1, 2, 3, 4 mL Route: Oral Period: 14 d	*100 neonatal Piglets (Duroc × Landrace × Large Yorkshire)	↑ Antioxidant capacity ↑ Intestinal villus height	↑ mRNA expression of intestinal tight junctions proteins (*occludin*, *ZO-1*, and *β-actin*) Modulate intestinal immune response ↑ IgG in serum ↑ Anti-inflammatory cytokines IL-4 and IL-10, in intestinal mucosa ↓ Pro-inflammatory factor IL-1β in intestinal mucosa	Li et al. (2019) [51]
*L. rhamnosus* GG	1 × 10^9^ CFU/mL Route: Oral Period: 15 d	18 weaned piglets *S. enterica* serovar Infantis 4, [5],12:i: challenge (10 mL oral dose, 1 × 10^10^ CFU/mL)	↓ Enteric infection Alter intestinal gut microbiota and keep homeostasis in intestine	↑ CD3-CD19-T-bet + IFNγ + and CD3-CD19-T-bet + IFNγ- cell subsets in the peripheral blood and intraepithelial cells in ileum	Zhang et al. (2019) [52]
Mixture of *L. johnsonii* L531*, B. licheniformis* BL1721 and *B. subtilis* BS1715	*L. johnsonii* L531, 10^8^ CFU/mL*; B. licheniformis* BL1721, 4 × 10^5^ CFU/mL *and B. subtilis* BS1715, 4 × 10^5^ CFU/mL) Route: Oral Period: 13 d	24 weaned piglets (Landrace × Large white) *S. Infantis* challenge (10 mL oral dose, 10^11^ CFU/mL)	Maintaining the intestinal mucosal barrier ↓ Intestinal cell death	↑ Claudin 1 and cleaved caspase-1 expression in ileum	Liu et al. (2019) [53]
*L. amylovorus,* strain P1 (LA), and *L. mucosae*, strain P5 (LB*), E. coli* Nissle 1917	8.0 log CFU Route: Oral Period: 7 d	55 gnotobiotic piglets *S. enterica* subsp. *enterica* serovar Typhimurium*,* strain LT2 challenge (oral dose, 6.0 log CFU/piglets)	↓ Clinical signs due to *Salmonella* infection	↓ Histopathological changes, the transcriptions of the proteins in intestine ↓ TNF-α, IL-10 in blood plasma	Splichal et al. (2019) [54]
*L. fermentum* and *P. acidilactici* (Commercial preparation)	(1.6 × 10^9^ CFU/g), mainly including 9.1 × 10^8^ CFU/g *L.* *fermentum* and 5.25 × 10^8^ CFU/g *P. acidilactici* Route: Oral Period: 28 d	*128 cross (Duroc × Landrace × Large white) weaned piglets	↑ Average daily gain ↑ Feed efficiency ↑ Growth performance, ↓ Inflammation ↑ Beneficial bacteria, ↓ Pathogens in intestine ↑ Production of short chain fatty acids in weaned pigs	↓ Concentrations of the serum proinflammatory factors IL-6, IFN-γ	Wang et al. (2019) [55]
*L. acidophilus* W37 + Inulin	5 × 10^9^ CFU/d/piglet + 0.114 g/d/kg body weight Route: Vaccine Period: 55 d	28 (Hypor × Maxter) newborn female piglets *S.* Typhimurium challenges (oral dose, 10^9^ CFU/piglet)	↑ Feed efficiency, fecal consistency	-	Lépine et al. (2019) [56]
*S. cerevisiae* S288c (Duan-Nai-An)	*S. cerevisiae* S288c 2.0 × 10^8^ CFU/mL, 10 mL/pig/d Route: Oral Period: 10 d	*108 crossbred piglets (Duroc × Yorkshire × Landrace)	↑ Intestinal health	↑ Plasmocytes and lymphoid nodule in gut ↑ Development of Peyer’s patches and germinal centers in gut	Zhaxi et al. (2020) [57]
*L. johnsonii* L531	1.0 × 10^10^ CFU/d Route: Oral Period: 18 d	18 weaned piglets (Landrace × Large White) of mixed gender *S.* Infantis challenge (10 mL oral dose, 1 × 10^11^ CFU/mL)	↓ Intestinal inflammation	↑ CD4^+^ , CCR6^+^ T cells in intestinal mesenteric lymph nodes Modulating T-cell responses and ER stress	Yang et al. (2020) [58]
*L. salivarius*	1 × 10^10^ CFU/g Route: Oral Period: 14 d	72 crossbred (Landrace × Yorkshiere × Duroc) piglets LPS from *E. coli* serotype O55:B5 challenge (200 μg/kg LPS intraperitoneally inject)	↑ Body weight, average daily gain ↑ SOD, CAT and GSH-Px in serum	↑ Tight junction protein ZO-1, Occludin and Claudin in intestine ↑ Serum IL-10 ↓ Serum IL-1β, IL-6, IFN-γ and TNF-α ↓ *TLR2* or *TLR4* expression in spleen and mesenteric lymph nodes	Sun et al. (2020) [59]
*L. plantarum* CJLP243, *L. fermentum* LF21 *L. salivarius* E4101 *L. paramesenteroides* KJP421 *B. subtilis* CJMPB957 *B. licheniformis* CJMPB283 (Multispecies probiotic formulation, MPF)	2 g/kg of MPF supplemented basal diet 10^11^ CFU/g (*L. plantarum*),10^9^ CFU/g (Rest probiotics) Route: Oral Period: 42 d	*80 growing-finishing pigs [(Landrace × Yorkshire) × Duroc]	↑ Body weight ↑Gut defense integrity Modulation of gut microbiota *Clostridiaceae*, *Lachnospiraceae*, and *Turicibacter* ↑ Jejunal ZO-1 expression	↓ mRNA expression levels of *IL-12* and *IL-1β* in jejunum ↓ Sterol regulatory element-binding transcription factor 1c (SREBP-1c), CCAAT/enhancer binding protein α -(CEBPα), acetyl coA carboxylase (ACC) and carnitine palmitoyl transferase 1β (CPT1β)	Kwak et al. (2021) [60]
*B. licheniformis *and *B. subtilis* mixture	4 × 10^9^ CFU/g Route: Oral Period: 42 d	*120 healthy crossbred piglets (Landrace × Large White)	↓ Jejunum crypt depth, ↑ Ileum, jejunum villus height ↑ Ileum villus height to crypt depth ratio	↑ Expression of E-cadherin in the colon ↑ Proinflammatory cytokines and *TLR-4* in ileum and colon	Wang et al. (2021) [61]
Heat-killed *L. rhamnosus*	1 × 10^9^ CFU/g Route: Oral Period: 28 d	*96 weaned piglets (Landrace × Yorkshire × Duroc)	↑ Growth rate ↑ Feed efficiency ↑ Apparent total tract digestibility ↓ Post-weaning diarrhea	↓ Concentrations of serum TNF-α, TGF-β1 and cortisol	Kang et al. (2021) [62]
*L. mucosae* LM1*,* *L. mucosae* LM1+ Mannnan oligosaccharide	10^9^ CFU/pig, 10^9^ CFU/pig + 0.1% mannan oligosaccharide Route: Oral Period: 35 d	100 weaned piglets (Large White × Landrace × Yorkshire) *E. coli* LPS challenge (oral dose, 100 μg/kg body weight)	↑ Feed efficiency ↓ ileal crypt depth	↑ Serum and ileal IgA ↑ Mucosal IgG	Li et al. (2021) [63]
*L. plantarum* N14 + pickle of Rakkyo	5%, 20%, 40% mixture, 10^7^ CFU/mL Route: Oral Period: 21 weeks	*20 healthy crossbred (Landrace × Yorkshire × Duroc) piglets	↑ Growth rate, body weight	↓ Phagocytic activity in blood ↓ Leucocytes count in the peripheral blood (5% and 20% mixture)	Islam et al. (2021) [64]
*E. faecium* R1	6.5 × 10^6^ CFU/g Route: Oral Period: 21 d	24 crossbred (Duroc × Landrace × Yorkshire) piglets LPS from *E. coli* serotype O55:B5 challenge (100 μg/kg LPS intraperitoneally inject)	↓ Diarrhea, feed to gain ratio ↓ LPS induced injury in liver and intestine ↓ Total nitric oxide synthase activity in liver ↑ Pancreatic antioxidant capacity ↑ Catalase activity in liver	↑ Glucan in plasma ↑ IL-1β in liver, mRNA levels of *villin* in jejunum and ileum ↑ *Bcl-xL* and *pBD-L* in ileum ↓ Prostaglandin 2 and malondialdehyde in liver	Zhang et al. (2021) [65]
*B. coagulans* commercial preparation	8 × 10^9^ CFU/g Route: Oral Period: 28 d	*90 piglets Duroc (Landrace × Yorkshire)	↑ Average daily gain, improves growth performance ↓ Diarrhea rate and diarrhea index ↓ Intestinal bacteria such as* Listeria, Micrococcus, Leuconostoc, Enterococcus*	-	Sun et al. (2022) [66]
*L. plantarum* LA, *P. pentosaceus* SMFM2016-WK1, *P. acidilactici* K and *L. reuteri* PF30	2.0 × 10^9^ CFU/kg Route: Oral Period: 2 weeks	90 male (Duroc × Yorkshire × Landrace) weaned piglets *Escherichia coli* and *Salmonella enterica* challenge (10 mL oral dose, 1.2 × 10^10^ CFU *E. coli* and 2.3 × 10^9^ CFU *S. enterica*	↑ Growth performance, diarrea incidence ↓ *E. coli* and *S. enterica* count in feces	~ Blood profile (WBC, neutrophil, lymphocyte, eosinophil, basophil)	Song et al. (2023) [67]
*B. licheniformis* CGMCC23776	10^9^ CFU/kg, 10^10^ CFU/kg Route: Oral Period: 28 d	216 weaning piglets (Duroc × Landrace × Large) LPS from *E. coli* challenge (1 mg LPS intraperitoneally injected)	↑ Growth performance, serum catalase activity, colonic major short-chain fatty acid, antioxidant capacity, ileal villus length ↓ Malondialdehyde concentration Modulate the colonic microbiota	↑ Serum IgA, IgG and IgM	Cao et al. (2023) [68]
*B. licheniformis* HJ0135	1 × 10^10^ CFU/kg Route: Oral Period: 28 d	120 weaning piglets (Duroc × Landry × Yorkshire) LPS from *E. coli* challenge (100 μg/kg LPS intraperitoneally inject)	Modulate saccharopine and allantoin from lysine and purine pathways ↑ Growth performance ↑ GSH-Px, SOD and T-AOC activities in serum	↑ Serum IgA and IgG ↑ Serum IL-10 ↑ Jejunal IgA, IgM and IL-10 ↓ Serum IL-6 and jejunum mucosal IL-1β and IL-18 ↓ LPS induced intestinal injury by regulating NLRP3	Yu et al. (2023) [69]

Abbreviations: ↑ Increased, ↓ Decreased, ~ Not changed, * Piglets were not challenged or infected with *E. coli*, *Salmonella* spp. and *Clostridium* spp., *LPS* Lipopolysaccharides, *IL* Interleukin, *Ig* Immunoglobulin, *TNF* Tumor necrosis factor, *IFN* Interferon, *CD* Cluster of differentiation, *TGF* Transforming growth factor, *CCR* Chemokine receptor, *TLR* Toll like receptor, *NF-kB* Nuclear factor kappa B, *WBC* White blood cell, *NLRP3* NOD-, LRR- and pyrin domain containing protein 3, *ETEC* Enterotoxigenic *Escherichia coli*, *SOD* Superoxide dismutase, *CAT* Catalase, *GSH-Px* Glutathione peroxidase

The aim of this review was to summarize the evidence for the use of beneficial microbes against diarrhea in piglets, focusing on common bacterial infections to assess how they reduce diarrhea and inflammation in the gastrointestinal tract of piglets. Moreover, this review aims to provide advanced knowledge to probiotic researchers, immunologists, swine nutritionists, and the probiotic industry to critically consider novel preventive approaches when applying or developing immunomodulatory probiotics to control diarrhea in piglets.

## Methodology

**Fig. 1:**
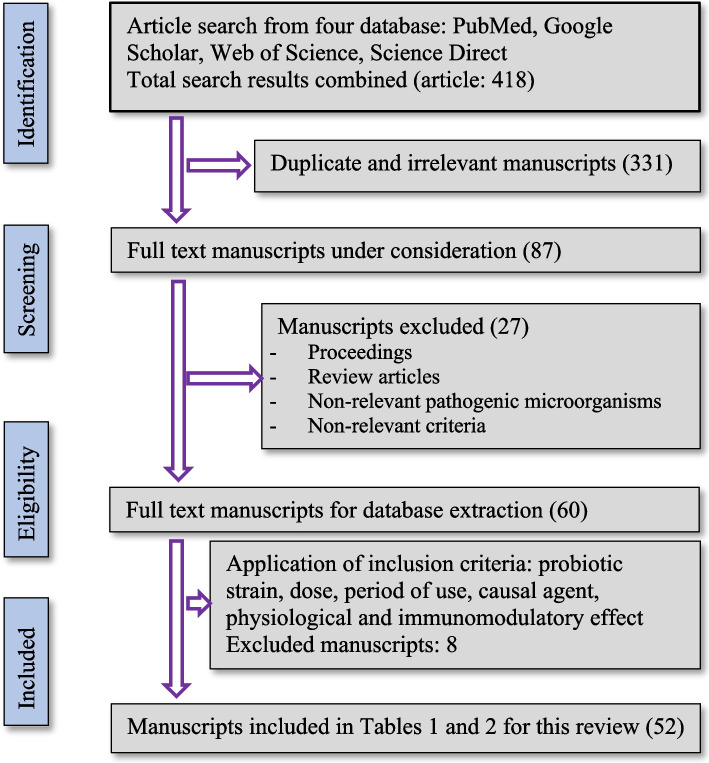
Diagram flow of manuscripts selection for this review

An electronic database was constructed based on published articles that reported the use of probiotics to control and/or treat bacterial diarrhea in piglets. We searched for articles published between January and March 2023. Articles were retrieved from PubMed, Google Scholar, Web of Science, and Science Direct databases using the following key words: probiotics, piglets, diarrhea, *E. coli*, *Salmonella*, *Clostridium*, lipopolysaccharides (LPS), and performance. The details of the article selection process of articles for this review are shown in Fig. 1.

## Common bacterial pathogens that cause piglet diarrhea

**Fig. 2:**
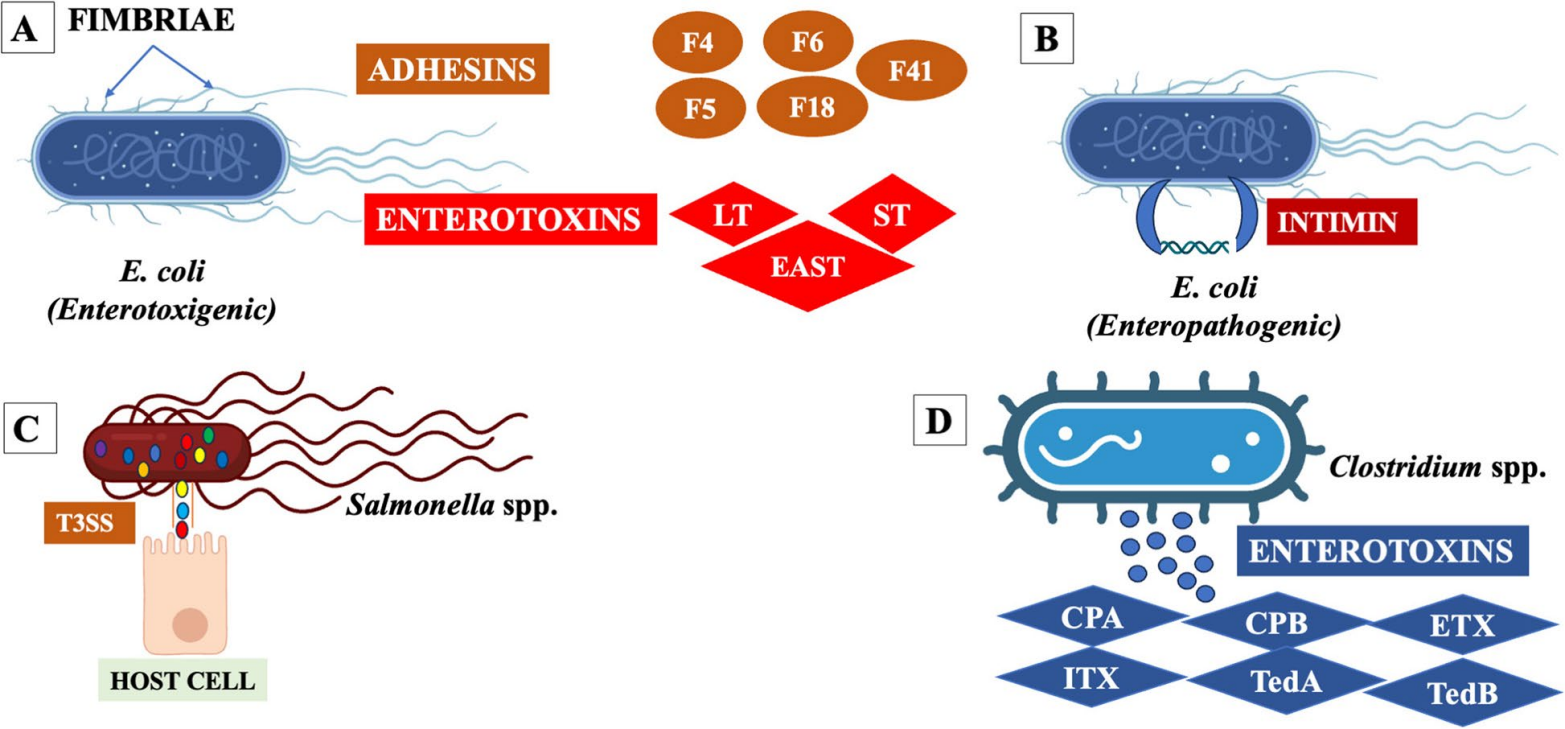
Visualization of **A**) *E. coli* (Enterotoxigenic, ETEC with fimbriae and enterotoxins), **B**) *E. coli* (Enteropathogenic, EPEC with intimin), **C**) *Salmonella* spp. with their effectors, and **D**) *Clostridium* spp. with enterotoxins. Abbreviations: F4, F5, F6, F18, F41, Fimbriae; LT, heat liable toxin; ST, heat stable toxin, EAST, Enteroaggregative heat stable toxin; T3ss, Three secretion systems; CPA, *Clostridium perfringens* toxin alpha; CPB, *Clostridium perfringens* toxin beta; ETX, *Clostridium perfringens* toxin epsilon; ITX, *Clostridium perfringens* toxin iota; TedA, *Clostridium difficile* toxin A, TedB, *Clostridium difficile* toxin B (Created with BioRender.com)

Bacterial pathogens are the most common cause of diarrhea in piglets [70]. The major bacterial pathogens causing diarrhea in piglets are *Escherichia coli*, *Salmonella* spp. and *Clostridium* spp. (Fig. 2)*.*

### *Escherichia coli* (*E. coli*)

*E. coli* are Gram-negative, facultative anaerobic, flagellated bacilli, and members of the Enterobacteriaceae family. They are the predominant etiological agents of a wide range of diseases in pigs, including neonatal and post weaning diarrhea, and are one of the major causes of death worldwide in neonates and weaned pigs respectively [71]. It causes diarrhea in swine, accounting for 56.2% of piglet cases, and is responsible for 24.7% of deaths due to diarrhea [72]. Based on the pathotypes of *E. coli* that can cause diseases in piglets, enterotoxigenic (ETEC) and shiga toxin producing (STEC) strains comprise two groups: 1) edema disease *E. coli* and enterohemorrhagic 2) enteropathogenic (EPEC) and extraintestinal pathogenic. Notably, the common categories related to enteric colibacillosis in piglets are ETEC and EPEC (Fig. 2A and B). Enterotoxigenic *E. coli* is one of the most common categories found in swine and includes different serotypes (different combinations of toxin and fimbriae). In general, diarrhea occurs during neonatal and post-weaning stages through ingestion of these bacteria, attaching to the mucus layer of the small intestine using hair-like structures known as fimbriae (F4, F5, F6, F18, and F41), whereas adhesin is involved in diffuse adherence (AIDA-I) and porcine-attaching effacing factor (Paa) is involved in non-fimbrial attachment. After colonization, ETEC produce enterotoxins that cause neonatal and post weaning diarrhea in piglets. In porcine neonatal diarrhea, most ETEC strains produce heat-stable enterotoxins that bind to the villous brush border guanylyl cyclase C glycoprotein receptor and intestinal crypt, inducing the production of cyclic guanosine monophosphate and leading to fluid and electrolyte secretion [73]. Heat liable toxins produced by ETEC bind to the cell surface and activate the adenylate cyclase system to induce cyclic adenosine monophosphate production. The upregulation of cyclic adenosine monophosphate activates the apical chloride channel and a basolateral Na/K/2Cl cotransporter, resulting in chloride secretion from enterocytes, reduced sodium absorption, and concomitant water loss into the intestine [74]. Excessive fluid loss due to diarrhea causes death eventually in piglets [73]. In post weaning diarrhea in piglets, ETEC strains contain fimbriae in their outer membrane layer, which are hair-like structures responsible for promoting the adhesion of ETEC to the mucosa of the intestine [75]. In addition, non-fimbrial adhesins including AIDA-I and Paa were expressed by ETEC strains, which facilitated the colonization of the intestinal tissue and produce heat-labile, heat-stable, and enteroaggregative *E. coli* enterotoxins. Enterotoxins have been shown to enhance the production of inflammatory cytokines and chemokines in the lumen and influence the expression of tight junction proteins in the intestines of piglets [76]. Inflammatory cytokines (IL-6, IL-17, and TNF-α), chemokines (IL-8, CXCL5, CCL2, and CCL8), and immune cells contribute to inflammation and intestinal damage during ETEC infection [31]. In addition, LPS, a major component of the outer membrane of bacterial cells, induces intestinal damage and diarrhea via an inflammatory response [77].

Enteropathogenic *E. coli* causes edema and diarrhea in piglets, producing and effacing lesions on intestinal epithelial cells and effacement of enterocyte microvilli [78, 79]. The EPEC pathotype can be categorized into two subgroups: typical and atypical. The EPEC possesses outer membrane proteins called intimin and the Paa which facilitate bacterial attachment to the translocated intimin receptor (Tir) of intestinal cells. Subsequently, the Tir-intimin interaction employed a non-catalytic tyrosine kinase (Nck) adaptor to activate the actin nucleation-promoting factor neural Wiskott-Aldrich syndrome protein which is responsible for inflammation and diarrhea [80].

### *Salmonella* spp.

*Salmonella* is a Gram-negative, motile, rod-shaped, facultative anaerobic bacterium belonging to the Enterobacteriaceae family that causes enteric diseases in pigs (Fig. 2C). Approximately 2,000 *Salmonella* serotypes have been recognized; however, few of them (*Salmonella enterica*, *Salmonella* Typhimurium and *Salmonella choleraesuis*) are responsible for most outbreaks in pigs. *Salmonella* spp. employs “effector” proteins using Type III secretion systems (T3SSs), which are molecular needle-like structures that allow invasion of effector proteins to the pig intestinal epithelial tissues [81]. The T3SSs secrete protein subunit, “Translocon” which can generate a pore in the membrane of host cell, resulting in virulence effector delivery into host cell, ultimately disrupting the intestinal epithelial cells [82] and dissemination of infection [83]. Upon entry into the host cell, *Salmonella* induces the expression of proinflammatory cytokines and chemokines through pathogen-related molecular patterns, such as peptidoglycan, LPS, and flagellin. Then, *Salmonella* can rapidly invades the intestinal lamina propria and causes acute inflammatory stimulus [84]. Inflammation in the intestine creates favorable conditions for the growth of *Salmonella* by altering the composition of the healthy gut microbiota.

### *Clostridium* spp.

The *Clostridium* genus comprise Gram-positive, rod-shaped, anaerobic, and spore-forming bacteria (Fig. 2D). *Clostridium perfringens* (serotypes A→G) and *Clostridioides difficile* commonly cause diarrhea in piglets. Among the seven serotypes (A→G) of *C. perfringens,* types A and C are the most common causes of diarrhea in piglets. Type A and C strains of *C. perfringens* produce the enterotoxins CPA, CPB, ETX, and ITX which are involved in creating lesions in the small intestine, and disrupting all layers of the intestinal wall, and inducing inflammatory responses [5]. Disruption of the intestinal wall facilitates the absorption of toxins from the intestine into the blood, leading to toxemia and death of piglets. In addition, toxigenic strains of *C. difficile* secrete two main toxins, TcdA and TcdB, which are involved in damaging the intestinal cell wall through the inactivation of Rho and Ras GTPases, activating inflammatory responses in the host, leading to an influx of cytokines and neutrophils that provoke intestinal wall and tight junction damage, ultimately leading to diarrhea in piglets [85].

## Effect of probiotics (immunobiotics) on pigs’ growth and health

Probiotics are expected to replace antibiotics as growth-promoting therapeutic agents. Thus, research on the use of probiotics is expanding. A single strain or combination of microbial strains have been used to control diarrhea and enhance the growth and health status of piglets (Table 2). Supplementation with probiotics in early life can improve piglet growth and healthy intestinal microbiota. As a result, early administration of probiotics can be a potential strategy to prevent diarrhea and restore microbial balance by establishing a microbiota balance after a transient drop in beneficial microbes, thereby contributing to the defense against disease-causing bacteria, improving nutrient absorption, and stimulating host immunity. Moreover, the morbidity and mortality of pigs decrease and growth performance and health conditions improve because of probiotic supplementation [86]. Several studies (Table 2) were performed to evaluate the effects of different probiotic strains on a wide range of health conditions and growth performances of piglets. Notably, different probiotic treatments using spores to survive and heat-killed microbes improve intestinal health and growth performance, and reduce diarrhea in young piglets. Probiotics have various beneficial health effects through different pathways such as reducing gut pathogens, increasing beneficial microbes in the gut, increasing nutrient absorption, and regulating immune responses. The administration of *Lactobacillus* enhances a healthier microbial fermentation profile by augmenting beneficial microbes and intestinal barrier function, which is evidence of better nutrient absorption [22, 32, 45]. For example, daily feeding of *Lactiplantibacillus plantarum* Lq80 (10^10^ cells) for 14 d to weaned piglets (21 days old) resulted in a significant reduction of *E. coli* and *C. perfringens* in feces and increased the *Lactobacillus* population in the intestine [32]. Another probiotic strain, *Limosilactobacillus reuteri* ZLR003 (2 × 10^9^ CFU/mL), was supplemented to 30-d old weaned piglets for 10 d, and it was found that *L. reuteri* ZLR003 contributed to healthy microbial fermentation and improved the beneficial microbes in the intestine [45]. Moreover, several *Lactobacillus* strains, such as *Lactobacillus jensenii* TL2937 and *L. plantarum* TL2766, were supplemented to piglets from 3 weeks of age for 14 weeks (until 17 weeks of age) [22]. The study reported no changes in plasma free fatty acids, glucose, triglyceride cholesterol, blood leukocytes, C-reactive protein, lymphocytes, phagocytic activity, or antibody levels between the *L. jensenii* TL2937 and *L. plantarum* TL2766 groups. However, *L. jensenii* TL2937 supplementation reduced the presence of K88, K99, and 987P ETEC strains in the feces whereas *L. plantarum* TL2766 reduced only 987P ETEC. Moreover, body weight was affected by *Lactobacillus* administration. These findings suggest that supplementation with *L. jensenii* TL2937 can improve the health and productivity of pigs. In another study, oral administration of a commercial preparation of a mixture of *Limosilactobacillus fermentum* and *Pediococcus acidilactici* (1.6 × 10^9^ CFU/g) to pigs weaned at 28 days old for a period of 28 d improved growth performance, daily gain, and reduced concentration of serum proinflammatory factors, IL-6 and IFN-γ [55]. Additionally, oral administration of 2 mL *Limosilactobacillus frumenti* JCM11122 (10^8^ CFU/mL) from 6–10 d prior to early weaning improves health promoting microbes by altering the intestinal microbial community, which leads to improved fatty acid and protein metabolism and also reduces disease-associated metabolic pathways. Improve intestinal integrity, and tight junction proteins (such as occludin, ZO-1 and claudin), and intestinal secretory IgA and IFN-γ levels by *L. frumenti* JCM11122 supplementation were reported [50]. Similarly, another probiotic strain, *Lactobacillus delbrueckii* CCTCCM207040 (5 × 10^8^ CFU/mL), was supplied at different doses (1, 2, 3, and 4 mL) to suckling piglets for 14 d, resulting in improved body weight, concentration of serum IgG, and anti-inflammatory cytokines with reduced incidence of diarrhea [51]. In a recent study, 18 days of intragastric supplementation with *Lactobacillus johnsonii* L531 (1 × 10^10^ CFU/d) in weaned piglets challenged with *Salmonella* Infantis reduced the severity of diarrhea and inflammation in the intestine and maintained intestinal homeostasis [58]. Probiotic supplementation in the form of heat-killed *Lacticaseibacillus rhamnosus* at doses of 0.1%, 0.2%, and 0.4% with diet in weaned piglets improved growth performance, modulated the immune response, and alleviated post weaning diarrhea [62]. On oral administration of *L. plantarum* N14 supplemented with Rakky pickles at different dose levels (5%, 20%, and 40%) in piglets showed that 5% or 20% dose level improved complement activity, phagocytic activity, and leukocyte count in the peripheral blood compared with those using a 40% dose or untreated controls. Moreover, the piglets that received *L. plantarum* N14-fermented Rakky pickle juice exhibited higher growth rates than controls [64].

Additionally, different probiotics from *Bacillus* strains improve growth performance and immunomodulation [61]. *Bacillus* spp. secrets enzymes that improve feed digestibility and promote animal growth. Providing *Bacillus subtillis* KN-42 (20 × 10^9^ CFU/kg feed) to weaned piglets for 28 d improved growth performance and average daily gain and reduced the diarrhea index and number of *E. coli* in feces [40]. In addition, the administration of a commercial preparation of *B. coagulans* (600 g/t) to weaned piglets for 28 d resulted in improved body weight, daily weight gain and reduced the incidence of diarrhea [66]. Wang et al. [61] showed that feeding a mixture of *Bacillus licheniformis* and *B. subtilis* (4 × 10^9^ CFU/g) for 42 d had a positive effect on piglet intestinal immunity by modifying the gut microbiota composition and concentration of microbiota-derived metabolites. Feeding complex probiotics (multi-species probiotic formulations), a mixture of different strains of *Lactobacillus, Bacillus*, *Saccharomyces* genera, with different compounds improve the health status and growth performance of piglets [33, 41, 49, 54, 60, 63]. Supplementation of 0.5% probiotics (*Lactobacillus acidophilus*, *L. plantarum*, *B. subtilis* and *S. cerevisiae*) with green tea to pigs for 28 d improved growth performance, and splenocyte production of IL-6 and TNF-α [33]. This may be due to the increased activity of different gastrointestinal enzymes, such as sucrase, lipase, protease trypsin, and chymotrypsin [50], and reduced intestinal permeability [53] by supplementation with probiotic bacterial species of the genera *Lactobacillus* and *Bacillus*, which leads to gastrointestinal peristalsis and promotes apparent digestibility [62, 87].

Furthermore, yeast can produce enzymes, such as amylase and galactosidase, which play vital roles in nutrient utilization and improve animal growth performance. Yeast can also regulate the intestinal microbial balance, strengthen the immune system, and improve animal health. Administration of *S. cerevisiae* S288c strain (2 × 10^8^ CFU/mL) fermented with egg white powder (Duan-Nai-An) for 10 d improved the intestinal structures and lymphoid tissues, and promoted improvements in the intestinal health in weaned piglets [57]. Feeding live yeast *S. cerevisiae* NCYC Sc 47 to nursery pigs for 45 d improved growth performance and body weight, and decreased *E*. *coli* concentration in pig feces [88].

## Probiotics against bacterial pathogens causing diarrhea in piglets

Recently, many researchers have evaluated the use of probiotics in prevention and treatment of various diseases of piglets [15, 89–91]. Evidence suggests that probiotics act as immune activators, particularly by boosting host immunity against pathogenic bacteria. Additionally, probiotics defend the intestinal tract by competitively excluding pathogenic bacteria that cause intestinal inflammation and diarrhea [15]. Moreover, probiotic use can reduce inflammation, restore barrier function, and mitigate the gut dysbiosis associated with diarrhea. Some probiotic genera (*Lactobacillus*, *Bifidobacterium*, *Bacillus*, *Enterococcus* and *Saccharomyces*) act as immunomodulators, regulate the proliferation and differentiation of lymphocytes (T and B cells), induce the secretion of cytokines and chemokines, and stimulate immune responses against bacteria in piglets [92]. The microorganisms that have been used as probiotics against pathogenic bacteria-induced piglet diarrhea are presented in Table 1 and 2.

### In vitro studies of probiotics against bacterial diarrhea in piglets

Some studies examined the effects of probiotics on bacterial pathogens using in vitro cell culture models (Table 1). Based on these findings, probiotic genera (*Lactobacillus *and* Bacillus*) may regulate immune response and antagonistic activity against bacterial pathogens, including *E. coli* and *S. enterica.* In the intestine, probiotic strains adhere to intestinal epithelial cells and modulate the intestinal immune system. The interactions between microbes and intestinal epithelial cells play a vital role in the regulation of several immunological functions in the gut. Thus, evaluating the anti-inflammatory activity of probiotic strains on porcine intestinal epithelial cells is useful for selecting immunobiotics [90]. Porcine intestinal epithelial (PIE) cells are used in vitro to evaluate the immunoregulatory mechanisms of immunobiotics against pathogens causing diarrhea in piglets, and several established PIE cell lines (PIE, IPEC-1, and IPEC-J2) have been used to study the potential probiotics against bacterial pathogens causing diarrhea in piglets (Table 1). Our research group used PIE cells because they are a useful cell line for studying inflammatory responses via toll-like receptors (TLRs) in epithelial cells. We tested the different strains of *Lactobacillus* and *Bifidobacterium* genera, such as *Lactobacillus jensenii* TL2937; *Ligilactobacillus salivarius* FFIG35, FFIG58; *L. plantarum* CRL1506, CRL681; *Limosilactobacillus reuterii* MEP221102, *L. rhamnosus* MEP221111; *L. salivarius* isolates*; L. fermentum* UCO-979C, CRL973; *L. delbrueckii* subsp. *delbrueckii* TUA4408L; *Bifidobacterium breve* M-16 V, *Bifidobacterium longum* BB536 [20–23, 27–29, 31] for studying their interaction with PIE cells, and found that different probiotic strains can differentially modulate the inflammatory response and produce different inflammatory factors in response to *E. coli* and *E. coli*-rotavirus superinfection. In vitro, strains CRL1506 and CRL681 regulated the gene expression of inflammatory cytokines (*IL-6*) and chemokines (*IL-8*, *CCL2*, *CXCL5*, and *CXCL9*) in ETEC-stimulated PIE cells. Baillo et al. [31] reported that ETEC challenged PIE cells treated with *L. plantarum* CRL1506 or CRL681 downregulate the gene expression of *IL-8*, *CCL2*, *CXCL5*, *CXCL9*, *A20* and *Bcl-3* by interference with inflammatory signaling pathways such as nuclear factor kappa B (NF-κB) and mitogen activated protein kinase (MAPK). Another study revealed that *L. jensenii* TL2937 downregulated the expression of *IL-6* and *IL-8* in PIE cells treated with ETEC [20] and in a co-culture of PIE and antigen-presenting cells stimulated with ETEC [22]. In addition, this strain led to the upregulation of negative regulators (*A20*, *Bcl-3* and *MKP-1*) of TLR4 in PIE cells, resulting in a marked decrease in inflammatory responses in PIE cells. Similar results were found for some *L. salivarius* isolates, which decreased the expression of *IL-6* in PIE cells challenged with ETEC [27]. While *L. fermentum* UCO-979C able to reduce inflammatory chemokines (*CXCL8*, *CXCL-10* and *CXCL-11*) in ETEC stimulated PIE cells by regulating the NF-κB pathway [28]. Notably, *L. fermentum* CRL 973 also reduces the expression of *CXCL-5* in ETEC stimulated PIE cells. Another study by our research group using PIE cells challenged with ETEC showed that *L. delbrueckii* TUA4408L inhibits the activation of MAPK and NF-κB pathways and the subsequent production of *IL-6*, *IL-8* and *MCP-1* and reduce the inflammation [23]. In addition, a study of different *Lactobacillus* and *Bifidobacteria* strains demonstrated that individual strains have different effects on the inflammatory response in ETEC-stimulated PIE cells. Particularly, *Bifidobacterium longum* BB536 and* Bifidobacterium breve* M-16 V strains reduce the expression of *IL-6*, *IL-8*, and *MCP-1* expressions in ETEC challenged PIE cells by modulating the MAPK and NF-κB pathways [21]. Similar to our results, *L. reuteri* LR1 decreased the expression of proinflammatory transcripts (*IL-6* and *TNF-α*) and increased the levels of anti-inflammatory cytokines (IL-10) in IPEC-1 cells after challenge with ETEC 0149:K91 and K88ac [24]. In addition, the findings of Zhang et al. [25] showed that *L. rhamnosus* GG could inhibit *S. enterica* serovar Infantis invasion in IPEC-J2 cells and *Salmonella* Infantis induced autophagy. *L. johnsonii* LJ202 and *L. reuteri* LR 108 completely inhibited the growth of *S. enterica* serovar Enteritidis DMST7106 in co-culture. *B. subtilis* CP9 strain was shown to increase anti-inflammatory granulocyte macrophage colony-stimulating factor and host defense peptides (such as mucin 1) and decrease the proinflammatory *TNF-α*, *IL-6*, *IL-8*, and *TLRs* mRNA expression levels in IPEC-J2 cells in response to *E. coli* challenge [26].

### In vivo studies of probiotics against bacterial diarrhea in piglets

#### Probiotics against *E. coli* pathogen

Oral supplementation with probiotics may prevent or improve diarrhea in piglets as summarized in Table 2. *Lactobacillus* species are used as feed additives and contribute to a balanced gut environment in various ways, such as protection against pathogens, improvement of intestinal health, and stimulation of immune responses. When *L. plantarum* CGMCC1258 is orally administered orally to ETEC-challenged piglets, it increases the expression of the genes for tight junction proteins (*ZO-1* and *occludin*), indicating an increase in strength of the intestinal epithelial barrier [39]. In addition, serum TNF-α production was markedly decreased in *L. plantarum *JC1 (B2028) treated animals [37]. A 28-d trial of oral administration of *L. plantarum* CJLP243 down regulated IL-6, TNF-α, and IFN-γ levels in serum and reduced acute inflammation of the gut after *E. coli* infection [35]. Another study by Sun et al. [59] reported that a newly isolated *L. salivarius* strain inhibited the expression of proinflammatory mediators (IL-1β, IL-6, IFN-γ, and TNF-α) in the serum and TLRs, such as *TLR2* and *TLR4* mRNA expression in the spleen and mesenteric lymph nodes after stimulation with LPS derived from *E. coli* serotype O55:B5. Moreover, supplementation with *L. salivarius *strain increased anti-inflammatory cytokines in the serum and epithelial tight junction proteins (claudin, occludin, and ZO-1) in the LPS-challenged pig intestine [59]. Improved mucosal immunity and IgA levels in the serum and ileum were also observed with a dietary supplement of* Limosilactobacillus mucosae* LM1and a mixture of *L. mucosae* LM1 and mannon oligosaccharides in LPS-challenged piglets [63].

Probiotics containing different strains or species have different efficacies in controlling or treating bacterial infection-related diarrhea in piglets [53]. *Bacillus* species can be used as feed additives in pellets because of their ability to survive under the low pH and harsh conditions in the gut. *Bacillus* species produce antimicrobial substances that kill pathogenic microorganisms and protect the intestines from pathogen invasion [93]. Yang et al. [44] reported that *B. licheniformis* (DSM 5749) and *B. subtilis* (DSM 5750) spores improved the integrity of the intestinal epithelial barrier by improving the jejunal ZO-1 protein expression and upregulated the intestinal *TLR4*, *NOD2*, *iNOs*, *IL-8* and *IL-22* and *IκBα* mRNAs expression and peripheral blood CD4^−^CD8^−^ T-cell in ETEC induced piglets. Furthermore, it was found that the oral administration of *B.* *licheniformis* (DSM 5749) and *B. subtilis* (DSM 5750) increased the production of CD4^+^Foxp3 T regulatory cells and CD4^+^IL-10^+^ T cells in the intestine to maintain the barrier integrity and protect the intestine from infectious agents in F4ab/acR^−^ (F4 fimbriae receptor negative) pigs challenged with an F4^+^ ETEC/VTEC/EPEC strain [42]. The same probiotic species with different strains namely *B. licheniformis* HJ0135 also has been used in a 28-d trial, where it was found to improve immune function and provide a positive effect by increasing the immunoglobulin (Ig) A concentration in serum and jejunum mucosal IgA and IgG, and decreasing serum IL-6 and jejunum mucosal IL-1β in response to *E. coli* LPS challenge [69]. Similarly, Cao et al. [68] reported that probiotic strain *B. licheniformis* GCMCC23776 enhanced the serum concentrations of IgA, IgG, and IgM in *E. coli* LPS-challenged weaned piglets.

Another notable study observed that the supplement of *Enterococcus faecium* R1 diminishes the injury in the intestine and liver of LPS-challenged piglets by increasing the glucagon in plasma and IL-1β in the liver. Moreover, the mRNA expression of *villin* in jejunum and ileum, and *Bcl-xL* and *pBD-1* expression in the ileum were upregulated by supplementation *E. faecium* R1. However, *E. faecium* R1 supplemented group reduced prostaglandin 2 and malondialdehyde content in the liver compared with that in the control group [65].

Additionally, using *S. cerevisiae* as a feed additive to *E. coli* challenged piglets activated the intestinal immune genes and improved body weight gain by increasing beneficial bacteria in the gut and reducing intestinal impairment [34, 48]. Supplementation with the *S. cerevisiae* CNCMI-4407 strain ameliorated the diarrhea and increased the concentration of IgA in the serum of piglets challenged with ETEC [38].

#### Probiotics against *Salmonella* pathogen

Oral administration of *L. plantarum* B298 to the *S.* Typhimurium-challenged piglets enhance the innate immune response by accentuating the immunoglobulin levels in the serum [43]. Similarly, the supplementation of *L. rhamnosus* GG controls enteric infection by restoring the gut microbiota balance and increasing the CD3-CD19-T-bet + IFN-γ + and CD3-CD19-T-bet + IFN-γ- cell population to maintain homeostasis in the intestine of *S. enterica* serovar Infantis-challenged in piglets [52]. The addition of *Bifidobacterium* strains such as, *Bifidobacterium animalis* subsp. *lactis* BPL6 and *Bifidobacterium longum* subsp. *infantis* CECT 7210 in the diet can improve intestinal immune function by enhancing intestinal intraepithelial lymphocytes in *Salmonella* Typhimurium challenge piglets [47]. A positive effect on the piglets health was observed by the oral supplementation of *B. cereus* var Toyoi by reducing the frequenting of CD8 + γδ T cells in the peripheral blood and gut epithelium in piglets challenged with *S.* Typhimurium [36].

Multi-strain probiotics can enhance intestinal immunity by modulating the immune responses in the intestine. The combination of *L. johnsonii* L531, *B. licheniformis* BL1721, and *B. subtilis* BS1715 improved the expression of tight junction proteins (claudin 1, caspase-1) in the gut of piglets challenged with *Salmonella* Infantis [53]. Furthermore, a study using the mixture of probiotics containing *Lactobacillus amylovorus* P1, *L. mucosae* P5, and *E. coli* Nissle 1917 for oral administration showed that the combination had a positive effect on reducing clinical signs and inflammatory responses in *S. enterica* serovar Typhimurium LT2-challenged piglets [54]. Another *L. acidophilus* strain W37, and inulin were assayed as vaccines for their efficacy against multidrug-resistant *Salmonella* Typhimurium-challenged piglets, and it was observed that feed efficiency and fecal consistency were improved the vaccination with *L. acidophilus* W37 and inulin supplementation [56].

#### Probiotics against mixed infection and *Clostridium*

Feeding a mixture of probiotic strains, namely *L. plantarum* LA, *P. pentosauceus* SMFM2016-WK1, *P. acidilactici* K, and *L. reuteri* PF30 to piglets challenged with *E. coli* and *S. enterica* resulted in beneficial effects on growth performance and reduced *E. coli* and *S. enterica* counts in feces [67]. Another study using a commercial probiotic *Lactobacillus* spp. product against *C. difficile* found a reduction in pathogen-induced mesocolonic edema; however, its effect on disease control in piglets remained unclear [46]. Therefore, the oral administration of probiotics has the potential to prevent and improve bacterial infections that can cause diarrhea in piglets. Most studies have focused on the use of probiotic strains against bacterial challenges in piglets, and more field studies on commercial pig farm conditions are necessary to determine the precise probiotic strains and dosages to control piglet diarrhea.

## Mechanisms of action of probiotics to alleviate piglet diarrhea

**Fig. 3:**
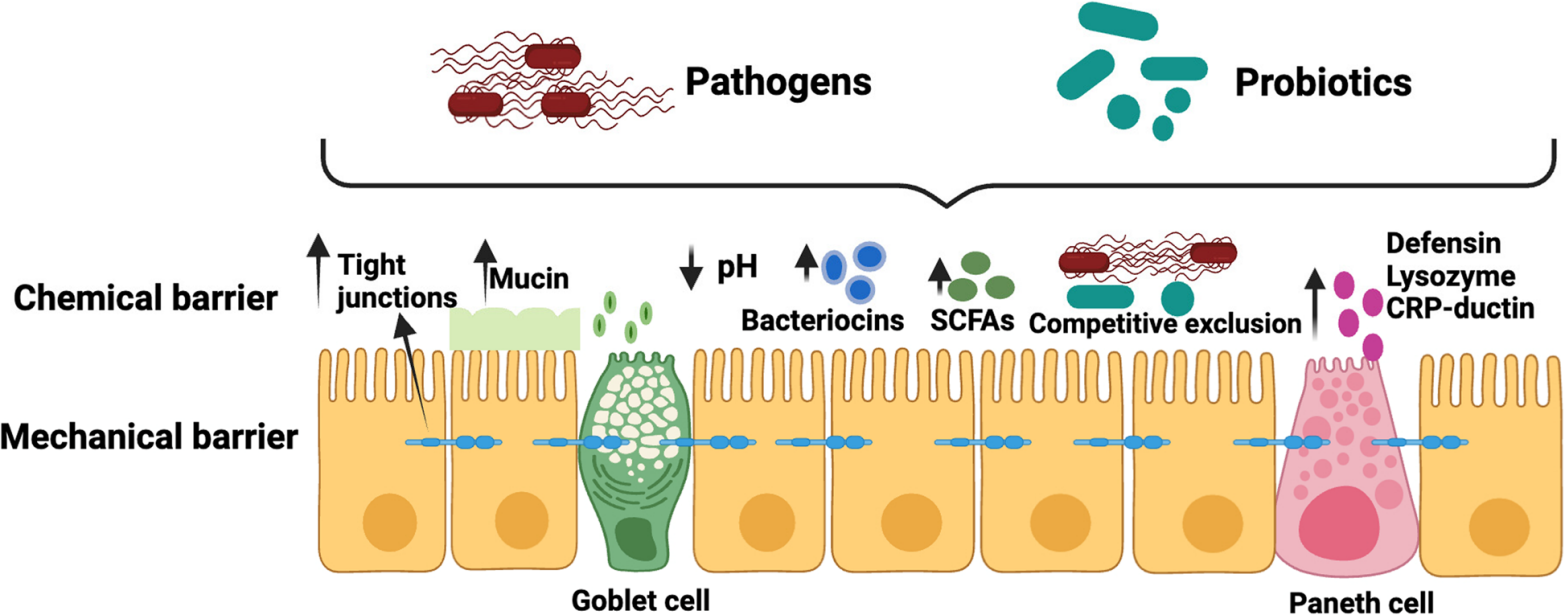
Interaction of probiotics on the intestinal barrier and its application in treating in piglets diarrhea. Probiotics alleviate diarrhea by regulating the intestinal microbial and mucosal barrier: 1) competitive exclusion of pathogen, 2) producing antimicrobial substance and neutralize toxins, 3) restore beneficial microbes, 4) upregulation of intestinal tight junction protein expression, 5) stimulate the secretion of mucin and peptides, and 6) reduce pH in intestine and helps to maintain normal intestinal permeability. Abbreviations: SCFAs, Short chain fatty acids (Created with BioRender.com)

**Fig. 4:**
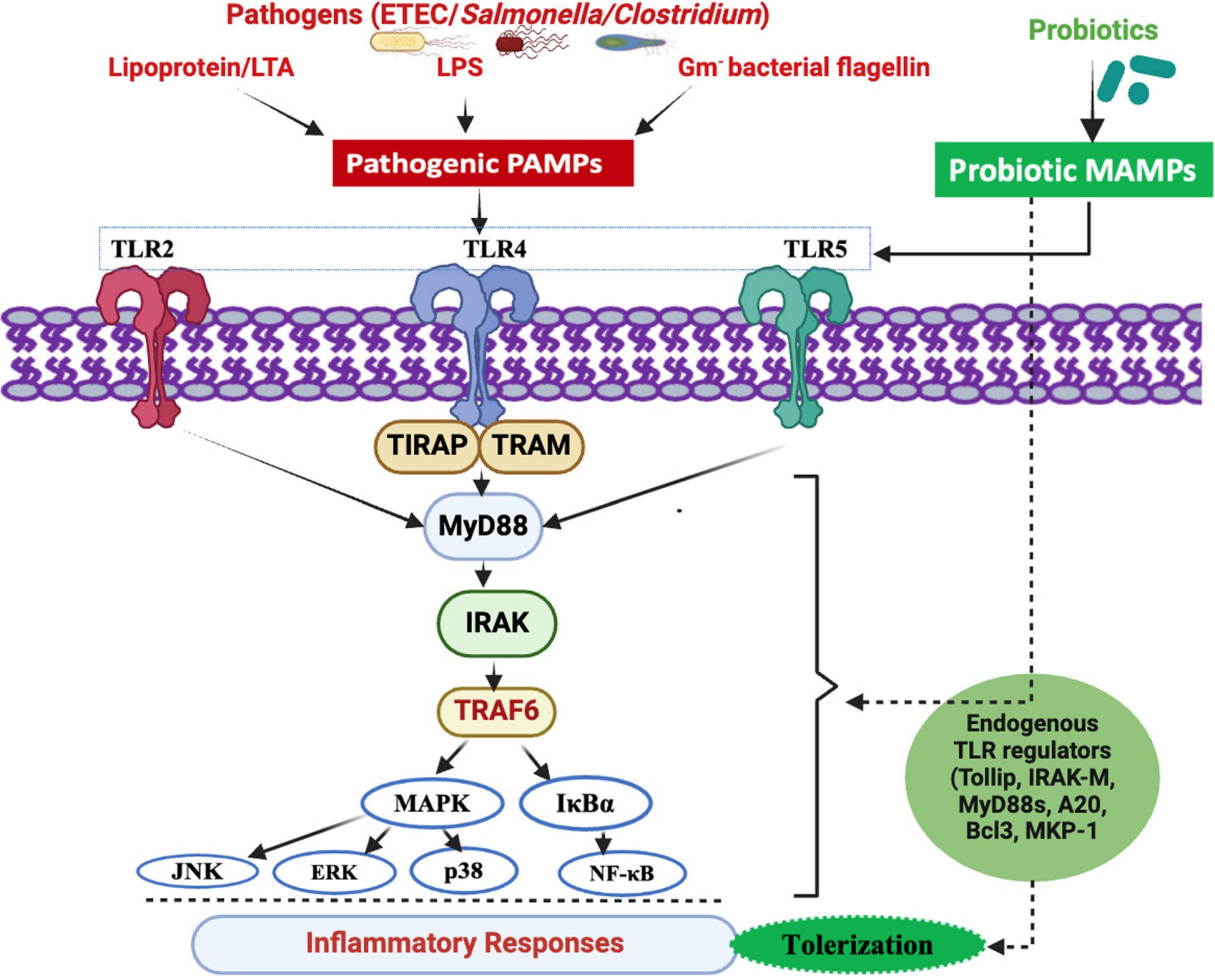
Probiotic modulation of gut associated immune system through TLR pathway regulation. Both pathogenic (red) bacteria (ETEC/*Salmonella*/*Clostridium*) and probiotic (green) bacteria can express similar/overlapping profiles of PAMPs/MAMPs (LTA, LPS, Flagellin) through a range of PRRs including TLR2, TLR4, TLR5. All of which can transduce immune activatory/inflammatory responses through activation of NFkB and MAPK signal pathways (indicated in black arrows). Probiotic-derived MAMPs (indicated as green), induce a suppressive/tolerogenic response via the induction of endogenous negative regulators to TLR signals (Tollip, IRAK-M, Myd88s, A20, Bcl3 and MKP-1) that inhibit NFkB and MAPK pathways. Abbreviations: LPS, Lipopolysaccharide; PAMPs, Pathogen-associated molecular patterns; MAMPs, Microbial associated molecular patterns; TLR2, Toll like receptor 2; TLR4, Toll like receptor 4; TLR5, Toll like receptor 5; TIRAP, Toll-interleukin-1 Receptor domain containing adaptor protein; TRAM, Translocating chain associating membrane protein; MyD88, Myeloid differentiation primary response gene 88; IRAK-M, Interleukin-1 receptor associated kinase M; TRAF6, Tumor necrosis factor associated factor 6; MAPK, Mitogen activated protein kinase; IκBα, IkappaB alpha; JNK, c-JUN N-terminal kinase; ERK, Extracellular signal-regulated kinase; p38, 38 kDa protein; NF-κB, Nuclear factor kappa B; A20, Tumor necrosis factor-α-inducible protein 3; Bcl3, B-cell lymphoma 3-encoded protein; MKP-1, Mitogen-activated protein kinase phosphatase-1 (Created with BioRender.com)

Although the precise mechanism of action of probiotics in the treatment of piglet diarrhea is not fully understood, two probable mechanisms have been proposed: regulation of the intestinal microbial barrier (Fig. 3) and the improvement of the immune system (Fig. 4).

### Probiotics and the intestinal barrier

The intestinal barrier acts as a major defense against pathogen invasion and maintains epithelial integrity and gut functionality. Newborn piglets develop diverse microbial communities in the gastrointestinal tract by consuming milk and exposure to the external environment. The dynamic balance of different gut microbiota acts as the first barrier to the gut. The mucosal layer serves as a protective barrier against pathogenic microorganisms, antigens, toxins, and other harmful substances. The mucosal layer acts as a protective barrier, which mainly comprises chemical and mechanical barriers [15, 94]. The chemical barrier consists of a mucosal layer and different digestive liquids (intestinal juices and enzymes) that are released by the mucosal epithelium of the intestine. Paneth and goblet cells, which reside in the intestine, play an important role in natural immune defense and support the intestinal barrier function [95]. Paneth cells produce different antimicrobial factors including, lysozyme, CRP-ductin, and α and β defensins [96] and these factors can disrupt the membranes or cell wall to kill the pathogenic bacteria and maintain homeostasis in the intestine [97, 98]. Additionally, intestinal goblet cells release mucin to form a protective layer of mucus at the top of the intestinal epithelial cells, preventing the entery of pathogenic bacteria into the epithelial barrier [99]. Mucins are glycoproteins that contribute to the maintenance of gut homeostasis and protect the intestinal barrier by interacting with the immune system. Interaction between intestinal microbes and host immune defense cell can subtly modulate intestinal barriers to prevent the invasion of pathogenic microbes and prevent inflammation in the intestine. This mechanical barrier is composed of various epithelial cells and intercellular tight junctions [15]. Intestinal epithelial cells and tight junctions effectively act as barriers to the invasion of bacteria and endotoxins from the intestine into the blood stream [100, 101].

Neonatal diarrhea occurs in piglets that do not receive colostrum and are born from non-vaccinated pigs. Some disease producing bacteria, such as *E. coli*, and* Clostridium* spp*. *can rapidly colonize the intestines of neonatal piglets, causing diarrhea, because the digestive and immune systems of piglets are not developed properly at this stage. After the neonatal stage, when weaning occurs, piglets tend to develop microbial diversity in the gastrointestinal tract because of abrupt changes in diet from milk to solid feed [102]. The gut microbial balance is formed based on feeding and maintains mutual relationships among different gut microbes, which acts as the first barrier of the gut against pathogens. During weaning, piglets experience stress that disrupts the intestinal barrier functions, which may result in the leakage of pathogenic microorganisms into the internal tissue layers, which in turn can cause inflammatory bowel diseases, such as diarrhea [103]. The consumption of probiotic bacteria involves the maintenance of intestinal barrier function; however, the mechanism by which probiotics maintain barrier function is not well understood. Probiotic supplements competitively prevent the adhesion of pathogenic microorganisms and exclude pathogens by producing antimicrobial substances in the intestine [61]. Probiotics stimulate paneth and goblet cells present in the intestinal epithelial layer, resulting in the production of mucins and antimicrobial substances that inhibit pathogen adhesion and kill pathogenic bacteria [104]. Moreover, probiotics can produce short-chain fatty acids in the gut, which contribute to lower pH levels in the intestine and enhance the gut barrier function by providing energy to intestinal epithelial cells [105], thereby inhibiting the growth of pathogenic bacteria [106]. Notably, probiotics can influence bacterial colonization by excluding or reducing pathogenic bacteria in the intestine and maintaining an optimal balance of the gut microbiota [103]. Probiotics secrete antimicrobial substances, such as bacteriocins and hydrogen peroxide to inhibit the adhesion of pathogens to the intestinal mucosa [107]. Probiotic supplementation upregulates intestinal integrity and expression of tight junction proteins that are damaged by pathogenic bacteria [108]. *Lactobacillus, Bifidobacterium, Bacillus*, and *Enterococcus* strains enhance intestinal barrier function in piglets challenged with ETEC [109]. In addition, probiotics, and their metabolites (such as organic acids, mannan oligosaccharides and β-glucan of yeast cell) may act as immune activators, which can stimulate the proliferation of T and B lymphocytes and the secretion of cytokine and chemokines and generate a series immune response [110].

In summary, the protection of the intestinal barrier in piglets may be improved by probiotic supplementation. However, the specific regulatory mechanism of probiotics on the intestinal barrier in piglets to alleviate diarrhea requires further research.

### Probiotics and the immune system

The immune system protects the host by segregating pathogenic and non-pathogenic microbes through different responses. Intestinal epithelial and gut-associated immune cells recognize molecules frequently found in bacteria via pattern recognition receptors (PRRs). PRRs are activated by specific pathogen-associated molecular patterns (PAMPs), which include various microbial components, such as LPS, peptidoglycan, flagellin, and bacterial DNA/RNA. Among the different families of PRRs, TLRs have been well studied and are expressed on diverse immune cells, such as B, macrophage, natural killer, dendritic, fibroblast, and non-immune cells, such as epithelial and endothelial cells [111]. TLRs play a prominent role in activating innate immunity and creating a link with adaptive immunity by modulating the functions of antigen-presenting cells and key cytokines [112]. Among the various TLRs, TLR4 recognizes and binds to LPSs, whereas TLR2 recognizes different PAMPs of pathogens (lipoprotein, peptidoglycans, lipoteichoic acids, zymosan, and mannan). TLR5 recognizes the flagellins in bacteria [113]. This compound is considered a potent inducer of inflammatory cytokines and chemokines. Although this response is considered the prime line of defense, prolonged and dysregulated responses may lead to tissue damage and dysfunction. In general, upon infection with bacteria or viruses, TLRs are activated and bind to their specific cognate ligands, resulting in the expression of peripheral membrane proteins, such as Toll-interleukin-1 Receptor domain containing adaptor protein (TIRAP) and Translocating chain associating membrane protein (TRAM) [111]. These adaptor proteins are involved in surveying the inner leaflets of the plasma-endosomal membrane [111]. TIRAP and TRAM can further recruit different negative regulators, such as myeloid differentiation primary response gene 88 (*MyD88*), interleukin-1 receptor-associated kinase M (IRAK-M), Toll- interacting protein, A20 and Bcl3 [97, 113]. IRAK-M heterodimerize with IRAK1- IRAK-2 and bind to *My88* and tumor necrosis factor-associated factor 6 (TRAF 6). Upon formation of this MyD88 adaptor complex which leads to activate IκB kinase and MAPK signaling pathways. After activation of IκB kinase and MAPK pathways resulting activation of JNK, ERK, p38 and NF-κB, which leading to induction of inflammatory cytokines [97]. Similar to TLRs, NOD-like receptors are another class of PPRs, which are cytoplasmic proteins that act as innate immune sensors to detect cytoplasmic pathogens [114]. Other types of PRRs include C-type lectin receptors, formaldehyde peptide receptors, retinoic acid inducible-like helicases, and intracellular IL-1 converting enzyme protease activating factor [115]. The supplementation of probiotics and their derived metabolites (such as organic acids, mannan oligosaccharide, and β-glucan of yeast cells) act as immune activators, which can trigger the dendritic cells, monocytes/macrophages, and lymphocyte, stimulating the secretion of a series of cytokines and regulating the immune responses [110]. Supplementation with probiotic bacteria can initiate responses via microbe associated molecular patterns (MAMPs). In fact, probiotics can tolerate immune signaling through different pathways, such as antagonism of pathogen-derived PAMPs, downregulation of PRRs, induction of suppressive cytokines, activation of antagonistic pathways, modulation of TLR negative regulators such as A20, Tollip, Bcl3, and MKP-1, and cross-regulation of TLR signaling [116]. *L. plantarum* CRL1506 and CRL681 can protect against inflammation-mediated damage in ETEC-challenged PIE cells by modulating the expression of the negative regulators A20, Bcl3, IRAK-M, and MKP-1 in the TLR signaling pathway [31]. The probiotic *L. jensenii* TL2937 upregulates the A20, Bcl3, and MKP-1 expression in PIE cells [97]. A20 is a zinc protein responsible for suppression of NF-κB signaling in response to TNF-α and microbial molecules LPS [117]. In addition, Bcl-3 protein acts as an inhibitor of NF-κB activity, whereas IRAK-M plays a crucial role in immune regulation through negative feedback loop by reducing the NF-κB and MAPK signaling [118]. In our earlier studies, using PIE cells exhibited a downregulation in the activation of NF-κB and MAPK signaling pathways and expression of several inflammatory cytokines and chemokines in ETEC-challenged PIE cells preventively stimulated with *L. jensenii* TL2937 [20], or *Bifidobacterium breve* M-16 V and *Bifidobacterium longum* BB536 [21]. Another recent study demonstrated that *B. subtilis* CP9 lowered the mRNA expression of *TLR2*, *TLR4*, and *TLR9* in IPEC-J2 cells co-incubated with CP9 and ETEC [30]. Moreover, pigs fed a diet with a probiotic mixture containing *B. subtilis* DSM 5750 and *B. licheniformis* DSM 5749 showed improved T cell regulation in the intestines of ETEC-challenged piglets [42]. Regulatory T cells play a pivotal role in the production of IgA antibodies, which play protective role against pathogens and toxins and prevent their invasion of the intestinal epithelium [28]. These findings demonstrate that the use of probiotic strains with immunomodulatory capacity could be an effective strategy for controlling or treating diarrhea in piglets.

Further research using multi-omics approaches may be useful to further investigate the mode of action of probiotic supplementation as well as their immunoregulatory capacity against bacterial diarrhea in piglets.

## Limitations of probiotics supplement

Research over the last decade has shown that probiotic supplements have positive effects on the health of pigs, but the conditions under which probiotics have been assessed are highly variable. Most studies have described the beneficial effects of probiotics rather than their adverse effects. Considering recently published data, probiotics may have a positive effect on the pathogenic bacteria responsible for diarrhea in piglets. Marked improvements, such as comparison with antibiotics and elimination of pathogens from the host, have not yet been reported. There are also some important differences in the experimental design of the studies, such as the age of piglets, treatment concentration, dosing amount, and methods, or other aspects, such as genetics, sanitary status, treatment days, or diets (Table 2). Furthermore, recently published articles have reported that probiotics can interact with commensal bacteria; however, their interactions have not yet been fully elucidated. Thus, understanding the interactions between probiotics and commensal bacteria is a major challenge for future research. Other strategic challenges are to determine their mechanisms, explicate which probiotic strain can work more specifically against which disease condition, and define the intake levels/doses needed to achieve the effects [119, 120]. An important limitation of probiotics is their ability to survive under during storage conditions. Thus, different environmental factors, such as temperature, humidity, acidity, and air should also be considered during probiotic storage. Otherwise, probiotic survival and the capacity to colonize the gut can be affected [121]. Regulations for the use of probiotics have been proposed by the European Food Safety Authority, and these beneficial microorganisms are considered zootechnical additives at the regulatory level [122]. Therefore, we may not have included or expected the same effects of probiotics as those of antibiotics. We can consider the use of probiotics as feed additives and combine them with other feed additives and management tactics with a more holistic approach [123].

## Conclusions

The use of probiotics and testing for their ability to prevent and treat bacterial diarrhea in piglets are increasing; consequently, they are being considered potential alternatives to antibiotics. We reviewed the research status of using probiotics to prevent or treat bacterial diarrhea in piglets and identified their potential regulatory mechanism from the perspective of intestinal barriers and the immune system. In contrast to antibiotics, probiotics commonly play a role in bacterial diarrhea by restoring the microecological balance in the intestine and regulating the function of the intestinal and immunological barriers. Different probiotic strains of the *Lactobacillus* group and *Bacillus*, *Enterococcus*, and* Saccharomyces* genera exert different health-regulatory effects to prevent or treat diarrhea caused by *E. coli*, *Salmonella*, and *Clostridium* in piglets by eliminating pathogenic microorganisms, producing antimicrobial substances, and degrading toxins, improving gut barrier function, and fostering proliferation, differentiation, and regulation of intestinal immune cells. More experiments (in vivo or in vitro) should be conducted to determine potential probiotics from normal weaned healthy piglets that can steadily colonize the piglet’s gut, improve gut mucosal barrier function, and activate the immune system to prevent diarrhea. In addition, the mechanism by which probiotic supplementation accelerates the maturation of intestinal microbiota or maintains homeostasis in the intestine during diarrhea in piglets warrants further investigation. Future studies should explore the specific effects of probiotic strains, address their viability and stability, and rationally design them to combat diarrhea in piglets.

## Data Availability

Not applicable.

## Abbreviation

aEPEC: Atypical enteropathogenic
AIDA-I: Adhesin involved in diffuse adherence
A20: Tumor necrosis factor-α-inducible protein 3
Bcl-3: B-cell lymphoma 3-encoded protein
Bcl-xL: B-cell lymphoma-extra large
CD4: Cluster of differentiation 4
CD8: Cluster of differentiation 8
CCL2: Chemokine ligand 2
CCL8: Chemokine ligand 8
CPA: *Clostridium perfringens* alpha toxin
CPB: *Clostridium perfringens* beta toxin
CRP: C-reactive protein
CXCL5: Chemokine (C-X-C motif) ligand 5
CXCL8: Chemokine (C-X-C motif) ligand 8
CXCL9: Chemokine (C-X-C motif) ligand 9
CXCL10: Chemokine (C-X-C motif) ligand 10
CXCL11: Chemokine (C-X-C motif) ligand 11
*E. coli*: *Escherichia coli*
EPEC: Enteropathogenic *Escherichia coli*
ERK: Extracellular signal-regulated kinase
ETEC: Enterotoxigenic *Escherichia coli*
ETX: *Clostridium perfringens* epsilon toxin
GTPases: Guanosine triphosphatases
IFN-γ: Interferon gamma
IgA: Immunoglobulin A
IgG: Immunoglobulin G
IgM: Immunoglobulin M
IκB: IkappaB
IL-6: Interleukin-6
IL-8: Interleukin-8
IL-17: Interleukin-17
IL-22: Interleukin-22
IPEC-1: Intestinal porcine epithelial cell line-1
IPEC-J2: Intestinal porcine epithelial cell line-J2
ITX: *Clostridium perfringens* Iota toxin
IRAK-M: Interleukin-1 receptor associated kinase M
JNK: C-JUN N-terminal kinase
LPS: Lipopolysaccharide
MAPK: Mitogen activated protein kinase
MCP-1: Monocyte chemoattractant protein-1
MKP-1: Mitogen-activated protein kinase phosphatase-1
mRNA: Messenger ribonucleic acid
MyD88: Myeloid differentiation primary response gene 88
Nck: Non-catalytic tyrosine kinase
NF-κB: Nuclear facto kappa B
NOD: Nucleotide-binding oligomerization domain
Paa: Porcine attaching factor
PAMPs: Pathogen-associated molecular patterns
pBD-1: Porcine beta defensin 1
PIE: Porcine intestinal epithelial
PRRs: Pattern-recognition receptors
Rho: Ras homologous
Ras: Guanosine-nucleotide binding protein
tEPEC: Typical enteropathogenic
TIRAP: Toll-interleukin-1 receptor domain containing adaptor protein
TNF-α: Tumor necrosis factor-α
TRAM: Translocating chain associating membrane protein
Tir: Translocated intimin receptor
T3SSs: Type III secretion systems
TcdA: *Clostridium difficile* toxin A
TcdB: *Clostridium difficile* toxin B
TLR: Toll-like receptor
TRAF6: Tumor necrosis factor associated factor 6
VTEC: Verotoxigenic *Escherichia coli*
ZO-1: Zonula occludens-1
